# A caring safety net or a broken shelter—Relatives’ experiences of specialized palliative home care: A national registry study

**DOI:** 10.1177/26323524261434624

**Published:** 2026-04-03

**Authors:** Annika Söderman, Fredrik Alm, Karin Blomberg, Helena Sjölin, Camilla Wall, Elisabeth Bergdahl

**Affiliations:** 1 School of Health Sciences, Faculty of Medicine and Health, Örebro University, Sweden

**Keywords:** dying, end of life, experiences, register, relatives, specialized palliative home care, survey

## Abstract

**Background:**

Cancer and circulatory diseases are overrepresented among the annual deaths in Sweden. Specialized palliative care (SPC) is considered complex, especially by cancer patients who have undergone long-term treatment. Relatives caring for a person with cancer within the home are faced with many demanding tasks.

**Purpose:**

To describe and map relatives’ experiences of the end-of-life care given in specialized palliative home care from a Swedish national perspective.

**Methods:**

A register study was conducted with a mixed-method design using qualitative and quantitative descriptive data from relatives (*n* = 563). Reflexive thematic analysis was used for the qualitative data, followed by a quantifying process inspired by Sandelowski.

**Findings:**

Two main themes were identified, with related themes and subthemes: “Home—a safe, dignified, and good place for dying” and “Home—a challenging, lonely, and unworthy place for dying.” As a place of dying during SPC, home provided both security and challenging events. It offered security for the family and upheld their dignity with the presence and support of competent healthcare professionals (HCPs). Conversely, challenging events emphasized how the relatives were unprepared for their loved one’s death and struggled to cope with the end-of-life situation. Some relatives felt that too much of the responsibility was left to them. However, the quantitative data showed that 99.1% of the relatives knew where to find urgent help during the last week of life, and the quantified qualitative data showed that HCPs were often available and supportive.

**Conclusion:**

Specialized palliative home care must strive to minimize challenging events that relatives experience during their loved one’s end of life and reinforce protective factors such as the presence of HCPs. As the end-of-life period is challenging for families, adequate support and competencies must be secured. Otherwise, there is a risk of increased family suffering and relatives having difficulties with grief after the death.

## Introduction

The use of specialized palliative care (SPC) has increased both nationally in Sweden and internationally. Its focus is to promote quality of life through provision of an integrated, holistic care to patients (and their relatives) among all ages and of which experience significant health-related suffering because of serious illness.^1^ The annual number of deaths in Sweden is approximately 94,000, and cancer and circulatory diseases are overrepresented. Nearly 22,000 patients need SPC each year.^2,3^ In Sweden, SPC should be performed by a multi-professional team with special competence and offered to patients with complex symptoms or with a life situation that entails special needs. Moreover, SPC in Sweden is given at home (often side by side with regular home care) or in a specific palliative care (PC) ward, either in hospital or in a hospice.^3^ SPC has been noted as more complex, especially by cancer patients who have undergone long-term treatment. These patients might have struggled for many years before being enrolled in specialized palliative home care (SPHC), and their relatives are likely to have fought by their side.^4^

It can be challenging to be an informal caregiver; relatives caring for a person with cancer within the home may feel poorly prepared and find that this work is complex and emotionally burdensome. They deal with these difficulties at the same time as dealing with their loved ones suffering from symptoms and distress.^5–7^ Relatives face physical, psychological, social, and existential challenges much of the time alone at home.^8^

Both cancer patients and relatives experience a range of unmet context-specific needs. One of the most common needs among relatives has been shown to be the need for information, for example, regarding the illness and the dying process.^8^ Relatives may also find it difficult to talk to each other within the family about dying.^9^

A recent Swedish national register study^10^ reported that approximately 1000 SPHC patients per year are transferred to inpatient care when near death. Of these, a large proportion die within 1 or 2 days after admission with common symptoms observed as severe shortness of breath, pain, and anxiety. These symptoms make it difficult for the patient to have a dignified death and are challenging for relatives to handle at home.

Further research is needed on relatives’ experiences before and during the period when their loved ones are dying. More knowledge can contribute to understanding what they and their loved ones are going through and help healthcare professionals (HCPs) and stakeholders understand the needs that may exist within SPHC. Furthermore, quantification of qualitative data in research is quite rare, yet numerical data on the distribution of relatives’ descriptions would help in drawing evident and broad conclusions from the results. The aim of this study, therefore, was to describe and map relatives’ experiences of the end-of-life care given in SPHC from a Swedish national perspective.

## Method

### Design

A register study was conducted with a mixed-method design inspired by Sandelowski et al.,^11,12^ using qualitative and quantitative descriptive data. The qualitative data were analyzed with reflexive thematic analysis^13^ and quantified.^11,12^

### Data collection

#### The Swedish Register of Palliative Care

The Swedish Register of Palliative Care (SRPC) is a nationwide quality register containing individually based information on expected and unexpected deaths in Sweden.^14^ The registry is well established and covers about 55%–60% of all deaths. In the event of a death, the HCPs at the unit where the death occurs fill in a digital form on the SRPC website. This form includes information on gender, age, date of death, hospitalization, place of death, diagnosis, symptoms, symptom management, and PC decisions. Within 3 months after the death, relatives are requested by mail from SRPC to complete a questionnaire with open-ended and fixed-choice questions regarding the evaluation of end-of-life (EoL) care and suggestions for improvement.

#### Participants

This study included relatives of patients with a cancer diagnosis who passed away in SPHC during 2021–2022, and who completed the SRPC questionnaire for relatives (Tables 1 and 2). The number of relatives who did not respond to the national survey was 6145.

**Table 1.** Respondents to the Swedish Register of Palliative Care questionnaire for relatives (*n* = 563) in terms of their relationship to the deceased patient.

Respondents	*n* (%)
Spouse	391 (69.4)
Child	138 (24.5)
Sibling	14 (2.5)
Parent	11 (2.0)
Friend	3 (0.5)
Other relative	6 (1.1)

**Table 2.** Demographic data of deceased patients whose relatives responded to the Swedish Register of Palliative Care questionnaire (*n* = 563).

Demographic data of deceased patients	*n* (%)
Sex	
Female	259 (46.0)
Male	304 (54.0)
Mean age	73.1
Diagnoses	
Cancer	563 (100)
Cardiovascular disease	15 (2.7)
Lung disease	10 (1.8)
Dementia	0 (0.0)
Stroke	4 (0.7)
Other neurological disease	0 (0.0)
Diabetes	4 (0.7)
Fracture	0 (0.0)
Multimorbidity	7 (1.2)
Infection	4 (0.7)
Other underlying disease	5 (0.9)
Symptoms*	
Pain	427 (75.8)
Severe pain	53 (9.4)
Respiratory secretion	247 (43.9)
Nausea	111 (19.7)
Anxiety	318 (56.5)
Dyspnea	112 (19.9)
Confusion	136 (24.2)

Data are given as *n* (%) unless otherwise specified.

* Reported by professionals with reference to the patient’s last week of life.

#### Survey questions from the SRPC

The survey questions from the SRPC that were used for this study are presented in Table 3.

**Table 3.** Qualitative and quantitative survey questions.

Qualitative survey questions	Quantitative survey questions
• Did you get the support you needed from the healthcare system before the death at the unit/in the team where your loved one died? • Was anyone present at the moment of death? (Both of these questions included a free-text field where the relative was invited to leave any additional comments)	• Did you feel that your loved one received the care they needed before coming to the ward/care team where they died? • Did you feel that your loved one understood that they were dying? • Did you receive counseling from a doctor who told you or helped you understand that your loved one was dying? • Did you know where to turn to receive emergency assistance (including at night, on a weekend, or on a holiday) for your loved one during the last week of life? • Did you know how to get in touch with the doctor who was responsible for your loved one? • Did you feel that your loved one received the care they needed from the ward/care team where they died? • How long before the death of your loved one did they lose the ability to express their wishes and participate in decisions about the care they would receive?

### Qualitative thematic analysis and statistical analysis

The data analysis began at a descriptive level with the qualitative data, and then moved to the quantitative data, which indicated sequential relationships in the data set.^12^ The qualitative data were converted into quantitative data in a quantified process^12^ and then analyzed through reflexive thematic analysis.^13^

The process and decision to quantify the qualitative data began with a national perspective, using visual qualitative data in the form of short drafts from the SRPC. One researcher (F.A.) entered the qualitative data into a Word document, using the purpose of the study to extract data relevant to the study. In the next step, two researchers (A.S. and F.A.) used one question based on the study aim as a guide in finding constructs in the data that could later be quantified^12^:

What constructs covering end-of-life experiences and the care given could be identified in relatives’ descriptions?

**Figure 1.**
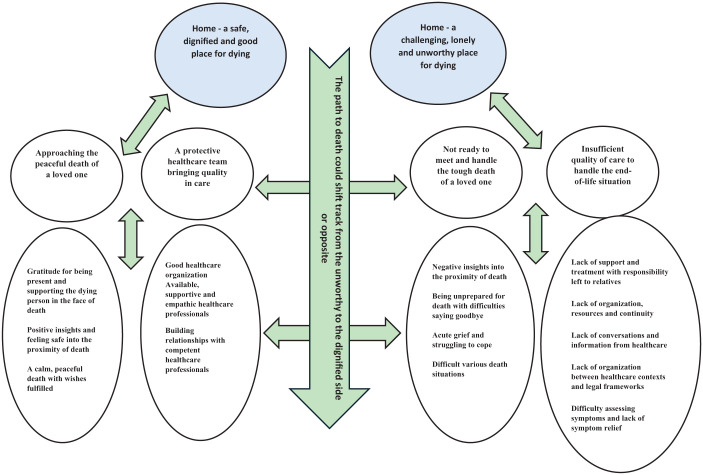
Thematic map over main themes, themes and subthemes.

The constructs were then collected in an Excel file and abstracted into preliminary labels (A.S.) that were intended to mean only one thing and therefore could be represented numerically.^12^ One of the researchers (A.S.) grouped these preliminary labels into preliminary subthemes based on their similarities and differences^13^ and then discussed the groupings with two of the other researchers (F.A. and E.B.) for calibration. Next, the whole research group (A.S., F.A., H.S., C.W., E.B., and K.B.) met for some refinement of the preliminary labels and subthemes. Some constructs were moved to different subthemes at this point. The first author (A.S.) then grouped subthemes with similar content into themes and identified two main themes. Following this, the whole research group met again to discuss and finalize the arrangement of the findings.^13^ Examples of the analysis are given in Table 4. Furthermore, to visualize how all themes were related, a thematic map^13^ was developed, see Figure 1.

**Table 4.** Example of the reflexive thematic analysis of qualitative data, themes on different levels together with quantified data and quotes from relatives.

Main themes	Themes	Subthemes	Quantified data	Quotes from registry data (items, constructs, variables)
Home—a safe, dignified, and good place for dying	Approaching the peaceful death of a loved one	Gratitude for being present and supporting the dying person in the face of death	Being present and supporting the dying person in the face of death	“*The last hours, I was by her side. When she took her last breath, we held each other’s hands, she looked at me and cleared her throat. Then it was over*.” (Spouse/partner of a 69-year-old woman)
			Gratitude for being able to be present at the death	“*So grateful to be by his side until the end*.” (Spouse/partner of a 78-year-old man)
		Positive insights and feeling safe into the proximity of death	Positive insights into the proximity of death	“*It was a nice moment of death. We (three out of four) siblings held our mother.*” (Children of a 79-year-old woman)
			Relatives feeling safe and without fear when death is close	“*It felt reassuring that we had the help of the staff at all times. You don’t feel alone in the difficult moments. My husband got help when he needed it*.” (Spouse/partner of a 63-year-old man)
		A calm, peaceful death with wishes fulfilled	A calm and peaceful death	“*When he took my hand, he became completely calm [. . .]. We talked for a few minutes. [. . .] then he became quiet, took three deep breaths and fell asleep peacefully*.” (Spouse/partner of a 53-year-old man)
			Fulfilled wishes at the end of life	“*Both nn and I were grateful to be together, and grateful that nn could be cared for in our home right until the end*.” (Spouse/partner of a 77-year-old man)
	A protective healthcare team bringing quality in care	Good healthcare organization	Good healthcare organization	“*. . . The entire course of care when we had ASIH [advanced home healthcare] was extraordinarily good in every way. Everyone in the team was incredibly friendly and caring and empathetic.*” (Spouse/partner of a 77-year-old man)
		Available, supportive, and empathic healthcare professionals	Available and supportive healthcare professionals	“*I’m very happy with how we were treated and the support we got from the time ASIH was connected. [. . .] Having the security of being able to call someone around the clock when a loved one is so ill is a huge security and makes it easier to cope*.” (Spouse/partner of a 50-year-old man)
			Receiving an empathetic response	“*[. . .] You are extremely sensitive in this situation, but their calm way of meeting us and explaining helped us*.” (Spouse/partner of a 70-year-old man)
		Building relationships with competent healthcare professionals	Building relationships	“*When my father died at home, the meetings with HCPs became very personal. We got to know the staff [. . .]. We felt a strong sense of security and a good feeling in your care of him*.” (Child of an 87-year-old man)
			Healthcare professionals with expertise	“*All the caregivers involved showed great compassion and competence. I felt confident that my mother was getting the care she needed.*” (Child of an 80-year-old woman)
Home—a challenging, lonely, and unworthy place for dying	Not ready to meet and handle the tough death of a loved one	Negative insights into the proximity of death	Negative insights into the proximity of death	“*It was difficult as a family member to interpret the signs from the dying person when there was no more verbal communication, and also to try to be as helpful as possible*.” (Child of a 78-year-old woman)
		Being unprepared for death with difficulties saying goodbye	Being unprepared for death	“*I’ve had great anxiety that I didn’t understand or wasn’t informed about how sick she was. IF I HAD KNOWN, I WOULD NEVER HAVE GONE HOME THE LAST NIGHT BEFORE SHE DIED.*” (Spouse/partner of a 66-year-old woman; capital letters in original)
			Difficulty saying goodbye	“*I couldn’t bear to be there at the time of death. I sat in the kitchen with a staff.*” (Spouse/partner of a 75-year-old woman)
		Acute grief and struggling to cope	Acute grief contributes to strong emotions	“*[. . .] I felt so bad that I could hardly cope. I still feel very bad and cry every day over having lost my dearest*.” (Spouse/partner of a 69-year-old woman)
			The struggle to find a coping strategy	“*I had no night cover at all during his last days of life, don’t know why I didn’t get it. It was very hard lately, should be available always, etc.*” (Spouse/partner of a 79-year-old man)
		Difficult various death situations	Unattended or unexpected death	“*We lived together around the clock for 3 months, and the last few days we watched at the deathbed, but for a 40-minute period on the last night she was alone, and then she went! Feels strange that it happened at that moment.*” (Child of an 83-year-old woman)
			A long and draining death process	“*He slept restlessly on the nights before. He commented that he felt like something was eating him up from the inside [. . .]*” (Spouse/partner of a 71-year-old man)
			A sudden death and/or a short death course	“*I, his wife, held his hand when he died, he couldn’t breathe, and he said ‘Help me’.*” (Spouse/partner of a 46-year-old man)
	Insufficient quality of care to handle the end-of-life situation	Lack of support and treatment, with responsibility left to relatives	Lack of support and treatment, or difficulty accepting them	“*Me and my three children wanted him to get care in a short-term home because he was so seriously ill, but I was RUN over by everyone. [. . .] It cannot be allowed to happen like this*.” (Spouse/partner of a 55-year-old man)
			Responsibility left to relatives	“*It took 3.5 months from nn’s brain haemorrhage (metastasis) until ASIH contacted us. In the meantime, we were referred to the emergency department and to the healthcare centre. Felt very unsafe with such a severe and advanced illness*.” (Spouse/partner of a 78-year-old man)
		Lack of organization, resources, and continuity	Lack of organization within the specialized palliative home care	“*ASIH is a medical team that is only supposed to take care of very sick people at the end-of-life, not people who need care as doctors and nurses do not have time to care if the sick person is in need of medication or control on other values*.” (Spouse/partner of a 75-year-old man)
			Lack of resources and continuity	“*He was anxious and responded poorly to the sedation. Then the nurse had to go to [the nearest city] and pick up and pack pumps—this took too long and he had a very hard time—even for us relatives.*” (Spouse/partner of a 77-year-old man)
		Lack of conversations and information from healthcare	Lack of conversations and information from healthcare	“*Ideally, provide written information about what is offered and how the care team is organized as soon as palliative care becomes relevant. If the care is going to take place at home, it’s difficult for relatives to know who is in charge of what*.” (Child of an 88-year-old woman)
		Lack of organization between healthcare contexts and legal frameworks	Lack of organization between healthcare contexts and legal frameworks	“*Difficult as a relative to distinguish between ‘home help’ from LAH [county-council-affiliated home healthcare], Home Service, Home Care. There are many people with different skills, information, personalities. [. . .] different activities within the region that may not always interact with each other.*” (Child of an 84-year-old woman)
		Difficulty assessing symptoms and lack of symptom relief	Difficulty assessing symptoms and lack of symptom relief	“*When it came to pain, it was difficult to assess, as ‘nn’ himself did not want any pain relief to a greater extent from the beginning.*” (Spouse/partner of a 67-year-old man)

Further information was then extracted from the qualitative data by treating preliminary labels in a quantitative way.^12^ The following questions guided the analysis in transforming qualitative data into quantitative numbers^11^: How often was each quantified data expressed by relatives in the data descriptions? What quantified data were most or least described by relatives?

**Figure 2.**
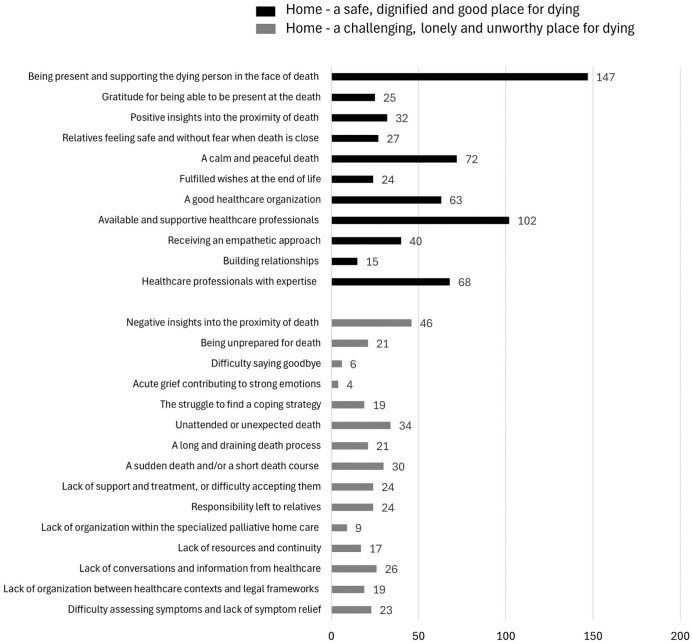
An overview of quantified data and their frequencies of occurrence in the palliative registry data during the study period.

Frequency sizes were then calculated to estimate the magnitude of the abstracted findings,^12^ visualized as a bar chart (Figure 2) to facilitate further interpretation.

Next, the qualitative and quantitative data sets were linked by configuration^11,15^ and combined at the interpretive level of research (data sets remained analytically separate from each other) to form a synthesis.

### Ethical considerations

The study was approved by the Swedish Ethical Review Authority (Reg. No. 2022-00740-01) and adhered to the beliefs of the Helsinki Declaration.^16^ To ensure the anonymity of the participants, data were disguised, and pseudonyms were used. Swedish legislation stipulates that before being registered in a quality registry as the SRPC, all patients are given the option to refuse participation in the registry and related research.

## Results

### Qualitative results and results from quantified data

The national perspective is presented in two main themes, themes and subthemes, along with quantified qualitative data and quotes that support the results (Table 4). In the text, “relative” refers to someone close to the dying person, and “loved one” refers to the person who was dying.

### Home—A safe, dignified, and good place for dying

#### Approaching the peaceful death of a loved one

##### Gratitude for being present and supporting the dying person in the face of death

The relatives described their loved ones living at home during the last days of life, with them participating in the care or being present at the time of death. Sometimes, the relative was alone with the dying person, but often the whole family or parts of the family were gathered. Many said that HCPs were also present. One relative who had accompanied their loved one in the ambulance said that the death had taken place there, during the journey. Several relatives stated that they were close by but asleep at the moment of death.

The relatives were thankful for being able to be present at their loved one’s death, and several expressed that this felt good. Some of them described that this was made possible by the HCP’s information and support. They said that information about the signs of an approaching death was helpful to them in assessing when the death was likely to happen.

The relatives appreciated having their loved one at home, as it allowed them to be there the whole time, making it possible for the death to happen in the home. One relative highlighted the importance of being able to express and scream out emotions before the HCPs came to the house, while another said that she would never forget the moment of death.

##### Positive insights and feeling safe to the proximity of death

Relatives experienced positive insights in the presence of death, thanks to HCPs and other family members, which made it possible for the loved one to be cared for at home. Being guided by HCPs was often expressed as bringing relief. The relatives appreciated knowing how much time was left and being able to have a relationship and conversations with their loved one until close to death.

Other positive insights concerned a symptom-free loved one and the maintenance of dignity, as the relatives were able to take care of their loved one after the death had occurred.

The relatives described feelings of security and fearlessness as their loved one approached death. These experiences were often related to having a healthcare team to call for help, which provided safety for both relatives and their loved ones. The relatives’ experiences of safety were underpinned by the presence of HCPs; being able to contact them when needed for support; and being able to talk to a doctor when desired.

Several relatives highlighted that when the SPC team became involved, this provided security. Being able to trust in healthcare was important, and being able to experience a calm, undramatic death where relatives felt prepared for death to occur.

##### A calm, peaceful death with wishes fulfilled

Many relatives said that their loved one had experienced a calm and peaceful death. They described examples of when their loved one just fell asleep, experiences of peacefulness, and good farewells. One relative said they had been talking to the dying person in the last few moments before death. The relatives described experiences of the passing as quiet and dignified. One said that her loved one wanted to be alone during the last days, but not on the last evening; then, he wanted her to be there all night long while being totally calm. Another person described a beautiful end, even though the relatives did not understand that their loved one would die very soon.

The relatives also described the fulfillment of wishes at the end of their loved one’s lives. These wishes sometimes came from the dying person, sometimes from the relative, and sometimes from both. The wishes included receiving more help when needed, the loved one being cared for at home until the end, with relatives or personal assistants present and involved in the care. But also, being able to be together as a family or with the partner at the EoL. Conversely, one relative described a loved one who had wished to be alone in the last hours before death.

#### A protective healthcare team bringing quality in care

##### Good healthcare organization

Many relatives felt that the healthcare organization was good, as their loved one’s healthcare team did their best to provide the optimal care. For example, they described doctors taking their time to make assessments and talk to their loved ones, a feeling of being involved in decisions, and getting the help they felt was needed. The HCPs had time for both the dying person and their relatives and provided the right information. The relatives appreciated being able to call for help at any time; they also appreciated it when the HCPs did that “little extra.” When the contact with SPC worked, the relatives were satisfied.

##### Available, supportive, and empathic HCPs

The relatives often described the HCPs as available and supportive. HCPs (a doctor, a nurse, or assistant nurse) were present in the home at the moment of death or arrived quickly after a call. Several relatives highlighted the security of having a staff member there whom they already knew from previous contact. Availability also included HCPs being there to answer questions. Frequent contacts were appreciated, together with conversational support. Many of the relatives felt gratitude for the support they received, as support made it easier to cope with the situation. Supportive qualities of HCPs were highlighted as being empathetic, understanding, and human. Thus, an empathetic response from HCPs was important to the relatives. This included nurses asking about their well-being, being able to meet the team counselor, and having a conversation with a HCP after the death had occurred. Other aspects highlighted were responsiveness, assistance in case of need, consideration and understanding, and a feeling of grace in the care of their loved one. The relatives described the importance of HCPs explaining what care actions were planned for their loved one. Thoughtfulness, a helpful appearance, and a respectful manner provided security for relatives in the difficult time of death.

##### Building relationships with competent HCPs

The relatives noted that the HCPs built relationships with them; for example, via personal meetings and keeping in touch by telephone. They said that the HCPs sat down with them and let them know they could reach out at any time. Some of the relatives described HCPs taking the time to explain what to expect and their options in the situation.

The relatives described respect, compassion, and professionalism as aspects underpinning good relationships with HCPs. They found value in the presence at the death of a previously known HCP.

Many relatives highlighted good care and the expertise of the HCPs within the team. Some of them said that the HCPs were fantastic and that they were impressed by their competence. Relatives appreciated it when they received explanations and medical assistance if necessary.

The relatives highlighted HCPs who took the initiative, were engaged, gave information about the phases and what was to come, and were friendly and nurturing. Many of the relatives had confidence in the team, and a professional approach provided security for the relatives. Some of them experienced the HCPs as safe and said that they guided them, answered their questions, and were knowledgeable about what could relieve symptoms.

### Home—A challenging, lonely, and unworthy place for dying

#### Not ready to meet and handle the tough death of a loved one

##### Negative insights into the proximity of death

The relatives experienced a range of negative insights when involved in a loved one’s death. They described difficulties in understanding that their loved one would die, and in knowing what to do for the dying person. Sometimes HCPs took the knowledge of relatives for granted, even though relatives themselves felt that they did not have the right knowledge. Occasionally, it was HCPs who did not understand that the person was dying. Relatives experienced strong emotions, a lot of anxiety, and felt unprepared for the tough times. Some were also not met with honesty in the situation, while others thought that remarks about the approaching death created confusion in the dying person.

Relatives noted that it was difficult to get information about a diagnosis and that there was often a delay before the loved one received full care. Requests that were not heeded created disappointment and anger. Furthermore, relatives described how difficult it was to witness the dying and that there were loved ones who did not yet feel ready for their lives to end. Experiences also concerned situations after the onset of death, as HCPs who failed to close the eyelids of the dead person.

##### Being unprepared for death and for the difficulties of saying goodbye

Several relatives had not accepted that their loved ones were going to die, and some found it difficult to accept help. Many felt unprepared, often because of the rapid dying process. A lack of acceptance by the dying person made communication in the family difficult, and for the relatives to gain understanding that death was approaching. The situation often became more apparent when palliative advanced healthcare at home (ASIH) was engaged. Some relatives had hope and faith that they would have more time together, while some had simply not observed the signs that death was near. Some relatives noted that there was sometimes a lack of preparatory information about what was going on, while others had received information but were unable to absorb it. However, HCPs mostly tried to help the relatives understand that death was near. Sometimes, the relatives were unsure if the dying person knew what was going on because of dementia.

Some relatives also described how hard it was to say goodbye to their loved one. One relative expressed the difficulty of thinking about the dying person’s situation due to their own anxiety. Another relative described how the death sequence made them so taken by the moment that they were unable to speak. However, difficulties saying goodbye were also due to more practical reasons; for example, during the COVID-19 pandemic, restrictions in care homes meant that the only contact allowed was through phone calls.

##### Acute grief and struggling to cope

A few relatives expressed strong emotions related to their grief; for example, by crying over the loss of a partner, or not being open to receiving help because of their own great anxiety. It could come as a shock when the relative really understood how close to death their loved one was. One relative explained that they could not remember their own state during the past week, but that as they tried to remember, they found themself crying, as they had a feeling of having wanted to be strong and accomplished.

The relatives testified to tough times, when anxiety and uncertainty were burdensome. Some described how the difficulty of understanding the situation—for example, not knowing what PC meant, created confusion. Relatives described how they had lacked guidance. They expressed that it was emotionally difficult to be helpful and try to understand the signs in the EoL process. One thing that made coping more difficult was when their loved ones did not want to talk about dying or lacked insight. There was also anxiety afterwards about not having had insight into the proximity of death, which led to not being present at the moment of death. Others testified that it had been difficult to witness an unpleasant death.

##### Difficult various death situations

Relatives described experiences of different severe deaths in relation to an unattended or unexpected death, a long and draining death process, or a sudden death and/or a short death course.

Several relatives described situations where their loved ones had died while the relatives were not in the room, were asleep, or had just left their visit. Sometimes, relatives could not get there on time. Some of the relatives said that they had not received the desired information that the death was coming, but others also believed that even having all the information would not help people understand what to expect. There were also descriptions of loved ones having been found dead by HCPs; sometimes the nursing staff were on site even if no relatives were present. One relative said that their loved one had died unexpectedly in an ambulance.

Some relatives had found that the process of death was draining not only for their loved ones but also for themselves. There were stories of unpleasant deaths. Relatives described endings that did not go well, where sedation or pain relief did not work. Sometimes, toward the end, the dying person exhibited difficult or altered breathing. The relatives highlighted the anxiety of their loved ones, and perceived feelings such as suffocation or drowning. The relatives said that HCPs could get tired, and that the uncertainty and responsibilities could be burdensome. For some relatives, the final period meant a feeling of being alone in the situation, missing the presence of HCPs.

Some of the relatives said their loved one’s death had been sudden, or that the death course had been short. This included symptoms suddenly emerging and changing the situation for the dying person. Sometimes, the rapid process meant that there was no time to make any contacts with HCPs or time to understand what was happening. Several relatives said that they were informed about the phases toward the end. However, some of them expressed disappointment over fighting for a long time for help that was then not needed because their loved one died so quickly.

#### Insufficient quality of care to handle the end-of-life situation

##### Lack of support and treatment with responsibility left to relatives

Some relatives had difficulty with accepting support, sometimes because of their own poor wellbeing, but in other cases they were not provided with support. They lacked EoL monitoring in the home, conversations with doctors, and close contact with healthcare. Some of them felt they were treated badly, for example when it came to understanding and conversations. The relatives described loved ones and themselves not being listened to, and requests not being complied with. Several relatives expressed that support became better after the initiation of SPC, as they felt that home care did not work so well or their support was non-existent.

Some relatives also shared stories about when the responsibility in the EoL situation was left to them. For example, when it had been up to them to contact the team, or aid officers who had not understood that the need for care changes rapidly at the EoL.

Relatives’ descriptions also emphasized a lack of cover and not being told how to best support their loved one. Some of them were employed in care work and were therefore knowledgeable about the field and said that they had been given too much medical responsibility, for example, in assessing when pain relief was needed. Cognitive problems or dementia in the dying person made it more difficult for the relatives if their loved one declined care at home.

##### Lack of organization, resources, and continuity

Some descriptions concerned shortcomings within the SPHC. For example, there was a desire for close contact initially, and it could take a long time before help was initiated if someone had to talk to the doctor. The relatives also expressed that there was a lack of time in relation to performing medical procedures in the patients who needed more caring interventions. The relatives had wanted more support in the final stages and after the death, for example by a counselor. Some of them said they were given the wrong phone number for the healthcare team, or leaflets about PC that created confusion.

The relatives described how a lack of cover meant they had to watch their loved ones themselves, leaving many of them exhausted. Proximity to resources was also lacking; for example, the lack of fentanyl patches in the medicine box meant that relatives had to travel to a distant pharmacy. A lack of oxygen or suction equipment in the home was also highlighted.

The relatives sometimes described substandard care; for example, when there was no time to care about the need for medication. It was desirable to have longer conversations when the loved one was diagnosed, as such conversations were perceived as difficult. Furthermore, relatives expressed that healthcare teams did not always talk to each other, and making different decisions was confusing for the relatives.

##### Lack of conversations and information from healthcare

The relatives did not always feel involved in the care and needed information about what awaited them at the EoL. They described a lack of conversations with HCPs; as talking to a doctor to get information about the time perspective, or talking to a nurse to ask why more pain relief was being given, or why no counselor contact took place despite this having been promised. One relative wished HCPs would have talked more about feelings with the dying person. Information about the close relative allowance had also, in some cases, come too late.

Not all relatives knew what PC meant. Some described not getting information about whether their loved one was in a palliative phase or about the illness itself. Some relatives had been forced to interpret the seriousness of the situation for themselves. Several relatives stated that the information was improved as the SPC team became involved.

##### Lack of organization between healthcare contexts and legal frameworks

The relatives expressed that it was difficult to know who was in charge of what (primary health center, home care service, district nurse, SPC) and to grasp how everything was organized. They described negative experiences related to the cooperation between different care contexts, such as between municipal home care and SPHC or between hospital healthcare and home healthcare. Sometimes, relatives lacked primary care as an intermediary between contexts.

Many relatives described shortcomings in relation to when their loved ones were sent home from the hospital and how the family “fell through the cracks.” They were referred to the emergency department and the healthcare center while waiting for ASIH, which sometimes could take several weeks to get. Care providers also disagreed about who was responsible for writing the referral to ASIH. The relatives noted that the municipality’s aid officers could have poor knowledge of PC needs, and there was sometimes a lack of understanding that home care was needed. Occasionally, the home care service did not know what care needs the person had when they came home.

##### Difficulty assessing symptoms and lack of symptom relief

The relatives stated that providing pain relief was a heavy responsibility to bear, and that the situation was made more difficult when there was no medicine pump, oxygen, or suction at home. They had found that the nurses’ assessments of their loved ones’ symptoms sometimes differed from the relatives’ own assessments. Symptoms that were described by relatives as being hard to alleviate were related to depression and anxiety, respiration, confusion, and pain breakthroughs, for example, if pain relief should have been given but was not. Relatives described how their loved ones experienced pain during nursing and transfers. Sometimes, the dying person could not tolerate the morphine patch. One relative described not understanding why the nurse gave their loved one more morphine, as the loved one was already highly sedated and the extra morphine would accelerate the death process.

### Quantitative results

The quantitative findings are presented in Table 5.

**Table 5.** Overview of results from quantitative survey questions from the palliative registry.

Did you feel that your loved one received the care s/he needed before coming to the ward/care team where s/he died?	
Yes, completely	323 (57.4)
Yes, partly	109 (19.4)
No, not completely	75 (13.2)
No, not at all	19 (3.4)
Don’t know	10 (1.8)
Not applicable	27 (4.8)
Did you feel that your loved one understood that s/he was dying?	
Yes, completely	380 (67.5)
Yes, occasionally/partly	121 (21.5)
Yes, but too late	10 (1.8)
No	17 (3.1)
No, but my loved one did not want to have more information than s/he received	13 (2.3)
No, as a close relative, I did not want the healthcare system to inform my loved one	3 (0.5)
Don’t know	19 (3.4)
Did you receive counseling from a doctor who told you or helped you understand that your loved one was dying?	
Yes, it was a good counseling session	409 (72.7)
Yes, but it was not a good counseling session	34 (6.0)
No	43 (7.6)
No, but from a different care provider	50 (8.9)
No, but I did not want more information than I received	15 (2.7)
Don’t know	12 (2.1)
Did you know where to turn to receive emergency assistance (including at night, on a weekend, or on a holiday) for your loved one during the last week of life	
Yes	558 (99.1)
No	5 (0.9)
Did you know how to get in touch with the doctor who was responsible for your loved one?	
Yes	528 (93.8)
No	35 (6.2)
Did you feel that your loved one received the care s/he needed at the ward/care team where s/he died?	
Yes, completely	498 (88.4)
Yes, partly	47 (8.4)
No, not completely	11 (2.0)
No, not at all	1 (0.2)
Don’t know	6 (1.1)
How long before the death of your loved one did s/he lose the ability to express his/her wishes and participate in decisions about the care s/he would receive?	
Hour(s)	138 (24.5)
Day(s)	283 (50.3)
Week(s)	52 (9.2)
Month(s)	13 (2.3)
Unable to make decisions for several months or more	3 (0.5)
Retained ability until the end of life	58 (10.3)
Don’t know	16 (2.8)

All data are given as *n* (%).

### Research synthesis

The results revealed relatives’ experiences of protective and non-protective care and caring factors near their loved ones’ EoL. The EoL path can be seen as a lottery, with much profit for most families but deep pitfalls for a few. Relatives who are unlucky may suffer severely for a long time with memories of a tough situation. Divergent experiences could also coexist within and across people, as end-of-life experiences can appear that are contradictory.

The relatives of a dying person hope for the best end, surrounded by a warm, caring safety net. As illustrated by the present results, this scenario comes true for most, but sometimes the challenges become too hard and too many, making the death process resemble a broken shelter. This carries the risk of deteriorating family health.

Many relatives have a clear path through the death process; however, a few of them have a less clear path, caused by a lack of information or previous experiences that could have helped them cope with this journey. The safety net of healthcare can become the only guard, and this may fail due to HCPs’ lack of competence and empathy, leaving relatives to feel completely unsupported. However, with protective factors in place, relatives can be helped to a safe harbor with maintained dignity.

The results suggest that protective factors include the guiding presence of HCPs who meet relatives with expertise and compassion, and with the aim of fulfilling EoL wishes. Conversely, unprotective factors include unmet needs, HCPs who cannot prepare relatives for a loved one’s death, and relatives being left alone and uninformed in an unbearable situation.

## Discussion

The home can be seen both as a dignified, good place for dying and as a challenging place for dying. This asymmetry in the results is not surprising as the Swedish expansion of PC nationally still is unequal. It has previously been reported that even if PC in western countries and in northern Europe has progressed and is developing, the availability of PC is far from equal and yet providing real challenges.^17^ However, this polarization can guide healthcare forward. If healthcare organizations dare to look at what factors lead to one or the other, strategies can begin to be formed for a more qualified care. To be able to enhance the care quality in PC, the process, performance, and outcomes of PC delivery must be assessed more regularly.^18^ Furthermore, previous research has shown that considering both place and death as definitive conflicts with the philosophy of modern EoL care, which emphasizes that dying is a process and an inevitable part of life.^19^ Keeping this in mind may help with acceptance that the home as a place for dying may not always be the best end scenario, and so it is important to consider how the whole death process develops and not only where the death is to take place.

The results showed that relatives and HCPs have a great role in the dying person’s last journey, indicating that their actions in different situations may have an impact on the person’s ability to maintain his or her dignity. To underpin dignity at life’s end and maintain quality of life is in line with the goals for good PC.^1^ However, these values can be difficult to achieve if the responsibility for care is left predominantly to relatives, as the present results imply sometimes happens. Participating in PC can thus be an overwhelming journey for relatives with inconsistent experiences, sometimes safe and sometimes challenging. Earlier research shows evidence of relatives’ deteriorating health when participating in PC. Relatives have reported high rates of psychological and existential distress, burden, and psychological morbidity. In addition, relatives report an alarmingly high number of unmet needs, with information being the central issue.^20^ Thus, having one constant professional contact, such as a PC facilitator, can help disseminate responsibilities and improve communication.^21^

The results showed that HCPs contributed to security for many relatives. Prior research shows that relatives providing home care can have the readiness to handle the EoL situation when the support and control over care in the home are working.^6^ Thus, SPHC services are influential for a sense of security in an uncertain and demanding situation.^8^ Providing the right support is also essential, as less support has been associated with lower perceived dignity.^22^ A previous review demonstrated that if dignity-conserving care is to be provided, it should reinforce family connectedness and foster coping strategies to control suffering.^23^

The results can be used as guidance to avoid a lack of dignity at the EoL, as some families suffered pitfalls that might have been avoided. Relatives with negative experiences were often due to a lack of information or being unprepared for their loved one’s death. Previous research shows likewise; throughout the care process, relatives can witness unbearable suffering and move between accepting dying and maintaining hope.^24,25^ On the other hand, other relatives felt they had thorough support, meeting empathetic HCPs with expertise, and learning from their positive experiences can give care organizations a direction forward. One recent integrative review,^26^ including studies partly from Europe, showed that family carers experience the transition from hospital to home as challenging and require preparation and coordination to prepare them to provide timely end-of-life care at home. Without a decided care plan, family carers felt unprepared, which negatively impacted on their experiences. Hence, the palliative approach must work through the whole care journey from getting a diagnosis to the person’s last breath. The word “palliative” is derived from the Latin “pallium,” which means mantle; this symbolizes enveloping and protecting the dying person.^27^ Our results show that the mantle remains intact for most people, but in some cases, the protection does not work, and holes develop in the fabric of the mantle. These are the holes that we must work to fill within SPHC.

In the results, the importance of HCPs’ expertise was highlighted by relatives, which may indicate that it is a protective factor for the well-being of relatives. If there is sufficient knowledge among the healthcare staff, it could mean a greater opportunity for them to teach relatives about the death process and help them be more prepared for the death. Being prepared or not was a crucial aspect described by relatives. Earlier research portrays that the exact nature of someone’s death cannot be predicted, but an important task for carers is to help relatives and their loved ones to think through different likely EoL scenarios to come to terms with dying. Thus, HCPs need to offer strategies to deal with not knowing what the end will be like.^19^ This is a very complex task, challenging HCPs within SPHC to their utmost. As the SPHC team cannot be on site at every moment, relatives can experience unmet needs.^28^ This highlights the necessity of safeguarding resources such as expertise, building empathic relationships, and fulfilling wishes within the SPHC.

Relatives often lacked the support of someone to keep vigil over the dying person, although many also experienced the presence of HCPs at critical moments. If modern PC is to meet relatives’ needs, SPHC must secure attendance and support from HCPs. A previous review^26^ highlighted that support during the patient’s dying stage had value for family carers to help them cope and manage beyond their grief. Family carers reported variations in their readiness for the patient’s death and sometimes experienced shock when death appeared. Moreover, research regarding promising interventions must be taken advantage of. One recently evaluated web-based intervention^9,29^ helped increase self-reflection and preparedness among relatives. Likewise, organizations need to be open to the use of additional professionals, such as death doulas, underpinning support to both relatives and HCPs. Such a caregiver can bring a compassionate presence to the bedside, engaging in existential care in similar ways to chaplains but without the religious focus. Further research is needed to understand how professional death doulas could contribute to PC.^30^

## Strengths and limitations

A strength of this study was that both qualitative and quantitative relatives’ responses from the national SRPC were used. As the qualitative and quantitative results were linked with a configuration approach,^11,12,15^ this provided a breadth of data that made it easier to answer the research question and form a synthesis. Providing numerical data about the distribution of observations helped draw conclusions and keep the focus on the whole picture, showing that quantification is a reasonable thing to do with qualitative data. However, it is important to acknowledge that qualitative findings are crucial to draw the right conclusions from evidence.^31^

Another strength of this study was the large and diverse qualitative material, which naturally encompasses a wide range of family experiences. This breadth occasionally resulted in perceptions that may appear contradictory, for example, feeling both informed and yet insufficiently prepared for crises or the imminence of death. While such variation may challenge the interpretive clarity of some subthemes, it also reflects the inherent complexity and ambivalence of families’ experiences, thereby providing a more nuanced and realistic understanding of preparedness in the context of severe illness.

It is important to have an awareness of the researchers’ ambition to distinguish and include data that dealt with SPC and to omit data that concerned home care as relatives’ descriptions concerned both. This meant researchers had a great responsibility while interpreting the data and during the construction of the results. The relatives’ descriptions were limited to short sentences, as the palliative survey only offers a small space to describe experiences. Consequently, relatives did not always answer the questions that they were asked; they answered more based on what they wanted to highlight. We interpreted this as meaning that the relatives used the small space in the survey in the way they wanted, to give the answers they wanted. This led to less in-depth answers, which even the relatives expressed had been to their detriment. However, even these short descriptions led to significant insights, although themes must be approached with caution. Not all the 563 relatives who responded to the survey gave qualitative descriptions; some of them only answered the quantitative questions. Thus, all relatives in this population are most likely represented in some way in this study. It must be noted that a power analysis for sample size calculation was not done, and researchers wanted to include all surveys that they had access to, and which were answered.

With consideration of selection bias, we want to highlight the large drop-out rate of non-responders, so certain types of respondents might be under-represented. The relatives who answered the survey may differ from those who did not, and therefore important voices can be missing that might have affected the results in another way. A randomized sample would have provided a more valid ground.^32^ However, responders and non-responders did not differ on the distribution between the sexes or the average age.

Since the results highlight strong responses about excellent care at the same time as strong responses about poor care, it could be that those with the strongest feelings about the EoL care situation were more likely to respond to the survey. The non-response may also represent people who did not understand Swedish, because the survey is answered in Swedish; although there is a translated version, they might not have received this. Regardless limitations, the national perspective offers a clear picture of what does and does not work in SPHC. It offers a bottom-up perspective derived from data, where a synthesis illustrates configuration of findings.^15^

The SQUIRE guidelines were used to report this study.^33^

## Conclusion

Home care organizations providing SPC must strive to minimize the challenges that relatives experience during the end of their loved ones’ lives and reinforce protective factors such as the presence of HCPs and empathic communication support. As the EoL is challenging for families, adequate support and competencies must be secured. This requires not just resources, but the right resources. HCPs who work in SPHC need to have sufficient knowledge to meet the challenging situations that families are affected by. If not provided safety, a family’s suffering will increase and can give rise to difficulties in dealing with grief after death. New professionals to support relatives might need to be considered to achieve a comforting EoL journey for families.

## Supplemental Material

sj-pdf-1-pcr-10.1177_26323524261434624 – Supplemental material for A caring safety net or a broken shelter—Relatives’ experiences of specialized palliative home care: A national registry study Supplemental material, sj-pdf-1-pcr-10.1177_26323524261434624 for A caring safety net or a broken shelter—Relatives’ experiences of specialized palliative home care: A national registry study by Annika Söderman, Fredrik Alm, Karin Blomberg, Helena Sjölin, Camilla Wall and Elisabeth Bergdahl in Palliative Care and Social Practice
